# Persulfate–Based Advanced Oxidation Process for Chlorpyrifos Degradation: Mechanism, Kinetics, and Toxicity Assessment

**DOI:** 10.3390/toxics12030207

**Published:** 2024-03-09

**Authors:** Youxin Xu, Chenxi Zhang, Haobing Zou, Guangrong Chen, Xiaomin Sun, Shuguang Wang, Huifang Tian

**Affiliations:** 1 Institute of Environmental Biotechnology and Functional Materials, School of Environmental Science and Engineering, Shandong University, Qingdao 266237, China; xuyouxin@wfust.edu.cn; 2 Shandong Engineering Laboratory for Clean Utilization of Chemical Resources, Weifang University of Science and Technology, Weifang 262700, China; sdzhangcx@gmail.com (C.Z.); A15910058268@outlook.com (H.Z.); guangrongchen2004@outlook.com (G.C.); 3 Environment Research Institute, Shandong University, Qingdao 266237, China; sxmwch@sdu.edu.cn; 4 Sino-French Research Institute for Ecology and Environment (ISFREE), Shandong University, Qingdao 266237, China

**Keywords:** chlorpyrifos, persulfate-based advanced oxidation process, degradation mechanisms, theoretical prediction, ecotoxicity assessment

## Abstract

Persulfate-based advanced oxidation process has been proven to be a promising method for the toxic pesticide chlorpyrifos (CPY) degradation in wastewater treatment. However, due to the limitation for the short-lived intermediates detection, a comprehensive understanding for the degradation pathway remains unclear. To address this issue, density functional theory was used to analyze the degradation mechanism of CPY at the M06-2X/6-311++G(3df,3pd)//M06-2X/6-31+G(d,p) level, and computational toxicology methods were employed to explore the toxicity of CPY and its degradation products. Results show that hydroxyl radicals (·OH) and sulfate radicals (SO_4_^•−^) initiate the degradation reactions by adding to the P=S bond and abstracting the H atom on the ethyl group, rather than undergoing α-elimination of the pyridine ring in the persulfate oxidation process. Moreover, the addition products were attracted and degraded by breaking the P–O bond, while the abstraction products were degraded through dealkylation reactions. The transformation products, including 3,5,6-trichloro-2-pyridynol, O,O-diethyl phosphorothioate, chlorpyrifos oxon, and acetaldehyde, obtained through theoretical calculations have been detected in previous experimental studies. The reaction rate constants of CPY with ·OH and SO_4_^•−^ were 6.32 × 10^8^ and 9.14 × 10^8^ M^−1^·s^−1^ at room temperature, respectively, which was consistent with the experimental values of 4.42 × 10^9^ and 4.5 × 10^9^ M^−1^ s^−1^. Toxicity evaluation results indicated that the acute and chronic toxicity to aquatic organisms gradually decreased during the degradation process. However, some products still possess toxic or highly toxic levels, which may pose risks to human health. These research findings contribute to understanding the transformation behavior and risk assessment of CPY in practical wastewater treatment.

## 1. Introduction

Chlorpyrifos (CPY) is a type of organophosphorus pesticides with broad-spectrum biological activity, which is widely used in various agricultural and horticultural crops as well as in households to combat biting and stinging pests [1,2,3]. In 2018, the annual production of CPY reached 28,600 tonnes, and the global demand for this insecticide has an increasing trend year by year [4]. According to the classification of the World Health Organization, CPY belongs to the second category of pesticides with moderate toxicity [5]. The half-life of CPY ranges from 10 to 120 days, depending on the surrounding environment [6]. Decades of monitoring researchers have reported that CPY levels in seawater, rivers, groundwater, and even rainwater in different countries exceed permissible levels [7,8]. The concentration of CPY can reach to 303.8 μg·L^−1^, far exceeding the environmental allowable limits in the United States and Canada [4]. Researchers have found that CPY can cause liver and gill tissue damage in carp with low concentrations in aquatic environments [9]. Furthermore, once exposed in CPY, humans and animals can also cause reproductive and developmental neurotoxicity risks [10,11,12,13]. Because of the persistence and potential ecological hazards of CPY in water environments, methods for its removal have been attracted considerable attention.

Different techniques such as adsorption, microbial degradation, and nanophotocatalysis have been proven effective in removing CPY from water environments [14,15,16]. However, these methods have certain limitations. Adsorption materials may pose environmental risks [17,18], microbial degradation processes are relatively slow [19,20], and nanophotocatalysis materials also face issues with recovery and reusability [21,22]. In recent years, persulfate-based advanced oxidation processes (AOPs) have gained increasing attention. This is because sulfate radicals (SO_4_^•−^) generated by persulfate have longer half-lives, higher redox potentials, and better selectivity compared to hydroxyl radicals (·OH) [23,24]. Shah et al. prepared Fe-ZnO using the sol-gel method and used it in activating the persulfate process; results demonstrated that the generation of high concentrations of SO_4_^•−^ and ·OH have strong oxidation effect on CPY degradation [25]. Xie et al. found that CPY presents a rapid removal rate and almost completely degraded with the ultrasound-activated persulfate process [26]. This study proposed that the removal mechanism of CPY could be divided into two possible degradation pathways. The first possible pathway is the α-dechlorination of pyridine molecular fragments under the attack of ·OH; the second possible degradation pathway is the breaking of the bond between phosphorus and oxygen connected to the pyridine ring by ·OH, resulting in the formation of chloropyridine and diethylthiophosphoric acid. Shang et al. explored the degradation effect of the combination of microwave and persulfate on CPY in soil and explored the oxidation of SO_4_^•−^ and ·OH, speculating on the process of oxidation, dealkylation, and dealkylation-hydroxylation [27]. However, due to the limitations in detection instruments, the existence of some short-lived intermediates during the degradation process may be not be detected. Therefore, the specific degradation pathways are still unclear and require further research and discussion.

Density functional theory (DFT) has been widely applied to study the chemical reaction mechanisms through elementary reactions [28,29]. DFT calculations can verify the existence of short-lived intermediates during the reaction process. Zhou et al. investigated the reaction mechanism and potential degradation products of CPY induced by ·OH in the atmosphere at the level of MPWB1K/6-311+G(3df,2p) //MPWB1K/6-31G(d) [30]. Theoretical studies indicate that ·OH addition to P atom, dehydrogenation of the -CH_2_- portion, and hydroxylation at the C atom in the pyridine ring are energetically favorable pathways for the reaction of CPY with ·OH. The main products of atmospheric oxidation are chlorpyrifos oxide, SO_2_, 3,5,6-tetrachloro-2-pyridinol, and O,O-diethyl phosphorothioate. Zhao et al. studied the degradation reaction mechanisms of two representative organophosphorus pesticides, namely mevinphos and monocrotophos, in the presence of ·OH in the atmosphere and water using quantum chemical methods [31].

The study aimed to establish the reaction rate constants, the degradation mechanism, and degradation products of CPY with SO_4_^•−^ and ·OH from persulfate using quantum chemical methods. With the degradation of CPY and the generation of the degradation products, it is anticipated that the eco-toxicity may made changes. Computational toxicology method is a valuable technique for the rapid screening of toxic substances, which can be used for the toxicity prediction of transformation products of organic pollutants in water environments [32,33]. In this study, the computational toxicology method is employed to predict the eco-toxicity of CPY and its products in water environments, aiming to estimate their potential environmental risks. The obtained theoretical results will help us better understand the degradation of CPY in sulfate-based AOPs and cover the shortage of experimental data in the CPY degradation processes.

## 2. Computational Methods

### 2.1. Mechanism Calculations

In this study, the quantum chemical calculations for all the geometry optimizations involved in the degradation process were performed using the Gaussian 16 program [34]. The geometric configurations of all the reactants, intermediates (IM), transition states (TS), and products (P) were optimized at the M06-2X/6-31+G(d,p) level. M06-2X has been proven to be a reliable method for calculating the transformation mechanisms and kinetics of organic pollutants [35]. Additionally, frequency analysis was performed on the optimized structures with the same method and basis set level to ensure that the structures corresponded to local minima and transition states with only one imaginary frequency. The rationality of the transition state structures was thoroughly confirmed by intrinsic reaction coordinate (IRC) analysis, which connected each transition state with its corresponding reactants and products [36]. Furthermore, to obtain more accurate energies, single-point energy calculations were carried out at the M06-2X/6-311++G(3df,3pd) level for all the structures. In order to account for the solvent effect in the entire system, the SMD model was employed [37]. This model is a novel continuum solvent model based on the self-consistent reaction field (SCRF) theory, where “d” stands for “density” and represents the utilization of the entire solute electron density without defining partial atomic charges. It is considered a more accurate method and has been successfully applied in some aqueous reactions involving SO_4_^•−^ and ·OH [38,39].

### 2.2. Kinetics Calculations

The kinetics results could be obtained from the KisThelP program, which is based on the Transition State Theory (TST) with Wigner tunneling correction [40,41]. Equation (1) shows the thermodynamic equivalent (k^TST^) using KiSThelP [40]:

(1) $$k^{TST}(T)=\sigma \frac{k_{b}T}{h}{\left(\frac{RT}{p^{0}}\right)}^{∆n}e^{-\frac{∆G^{0,\neq }(T)}{k_{b}T}}$$

where σ is the reaction path degeneracy, k_b_ is the Boltzmann’s constant, T is the temperature, h is the Planck’s constant, and ΔG^0,≠^(T) represents the standard Gibbs free energy of activation for the considered reaction.

When the reaction involved precursor complex (PRC), the reaction rate constants were calculated using the following formula [42]:

(2) $$R_{1}+R_{2}\begin{array}{c} \overset{↔}{K_{eq}} \end{array}\mathrm{PRC}\begin{array}{c} \vec{k_{1}} \end{array}P$$

k = K_eq_ × k_1_ (3)

where K_eq_ represents the equilibrium constant for fast pre-equilibrium between the reactants and k_1_ represent the unimolecular reaction. The thermodynamic expression of the K_eq_ is employed in KiSThelP, as described in Equation (4) as follows [40]:

(4) $$K_{eq}=e^{-\frac{∆G^{0}(T)}{RT}}$$

In the formula, ΔG^0^(T) is the associated standard reaction Gibbs energy at temperature T and R is the ideal gas constant.

### 2.3. Eco-Toxicity Assessment

The toxicity assessment of CPY and its degradation products were determined using the quantitative structure-activity relationship (QSAR) based Ecological Structure Activity Relationship (ECOSAR V2.2) predictive model [43]. Fish, dephnia, and green algae were selected as aquatic organisms to evaluate acute and chronic toxicity risks. The acute toxicity for fish and daphnia was determined using the median lethal concentration (LC50), which means 50% lethal concentration of fish and daphnia after 96 and 48 h exposure, respectively. The acute toxicity for green algae was determined with the median effect concentration (EC50), which means 50% effective concentration of green algae after 96 h exposure. The chronic toxicity value (ChV) was used to reflect the chronic toxicity for three aquatic organisms. In addition, the toxicity evaluation software tool (TEST V5.1.1) was used to assess the bioaccumulation factor, developmental toxicity mutagenicity of CPY, and its degradation products [44].

## 3. Results and Discussion

To validate the computational model, the bond lengths of the CPY molecule at the M06-2X/6-31+G(d,p) level were calculated (Table S1). The calculated results are in good agreement with experimental values, with deviations of the calculated bond lengths from the experimental values within 5% [45]. These results demonstrate the reliability of the computational level and the CPY model.

### 3.1. CPY Structure Analysis

**Figure 1:**
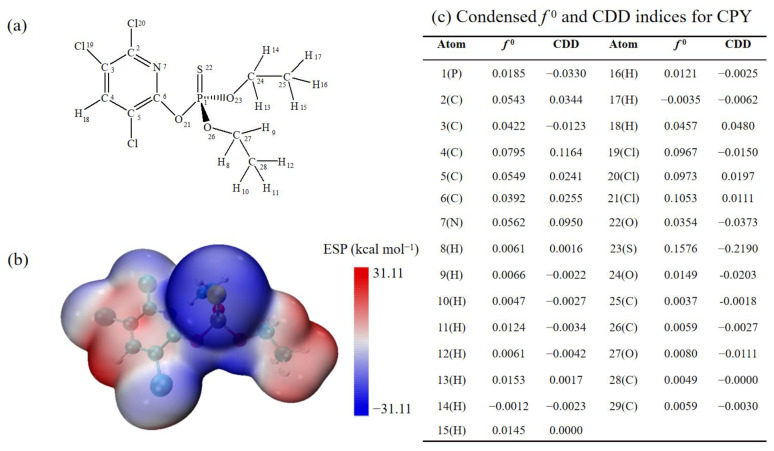
(**a**) The structure of Chlorpyrifos. (**b**) Molecular surface electrostatic potential distribution diagram of CPY. (**c**) Condensed *f* ^0^ andCDD indices for CPY.

In order to have a clear and description, all atoms have been labeled, and the structure of CPY is shown in Figure 1a. It can be seen that CPY contains a pyridine ring, a P=S bond, and two ethyl groups. To predict the reaction site, the average localized ionization energy of CPY was calculated, and the results of Fukui function and dual descriptor were analyzed, as shown in Figure 1b,c.

Figure 1b shows that the bluer the color, the more negative the electrostatic potential; and the redder the area, the more positive the electrostatic potential. Additionally, when the molecular surface electrostatic potential value became smaller, the electron reactivity became stronger, resulting in electrophilic and radical reactions. Figure 1b also shows that the electrostatic potential map of CPY confirmed that the P=S bond region is a relatively electron-deficient active region, which is easily attacked by active radicals. The pyridine ring and ethyl regions of CPY tend to be attacked by nucleophiles.

Figure 1c shows the Fukui function (*f*^0^) and dual descriptor analysis (CDD). The region with the highest *f*^0^ is the easiest one to be attacked by active radicals, and for the CDD, the region with the largest negative value is the easiest to be attacked by electrophiles [46]. The most prominent area of *f*^0^ is located at S_22_, indicating that these sites should be easy to be attacked by active radicals. In addition, according to the CDD, S_22_ is the most negative site, which means that the site should be the easiest to be attacked by electrophiles. Therefore, both methods indicate that the P=S bond in CPY is the most vulnerable to be attacked by ·OH and SO_4_^•−^.

Furthermore, ·OH and SO_4_^•−^ have strong oxidizing properties, and they can easily undergo addition reactions with unsaturated bonds and abstraction reactions with H atoms [28,29]. Therefore, it is considered that the addition reactions of radicals with the P=S bond and pyridine ring, as well as the abstraction reactions with H atoms.

### 3.2. The Reaction of CPY with the ·OH

**Figure 2:**
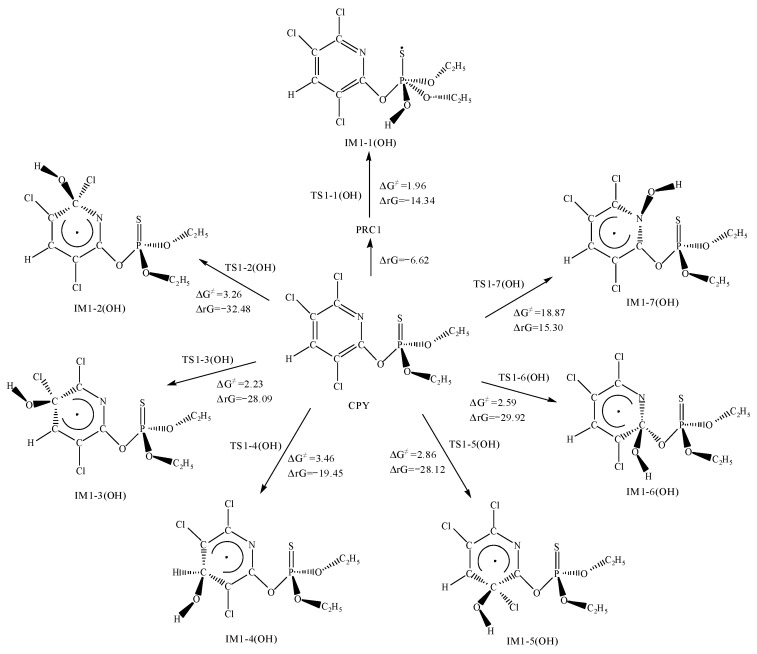
The Gibbs free energy barrier ΔG^≠^ (kcal·mol^−1^) and the Gibbs free energy change ΔrG (kcal·mol^−1^) at 298 K for ·OH addition pathways with CPY.

**Figure 3:**
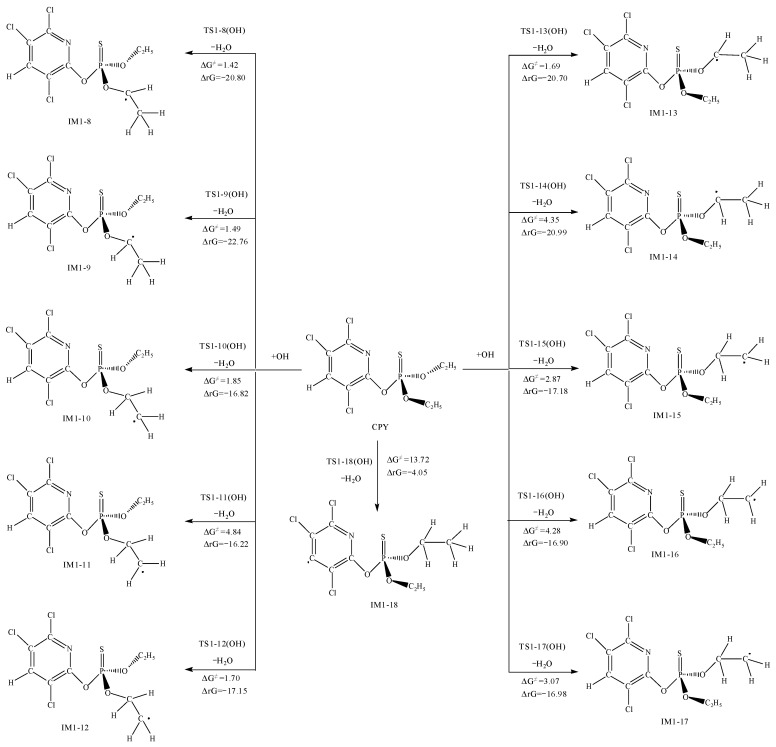
The Gibbs free energy barrier ΔG^≠^ (kcal·mol^−1^) and the Gibbs free energy change ΔrG (kcal·mol^−1^) at 298 K for ·OH abstraction pathways with CPY.

Figure 2 and Figure 3 show the addition and abstraction reactions of CPY with ·OH, respectively. The optimized chemical conformations of TS for CPY with ·OH are shown in Figure S1. Here, ΔG^≠^ represents the Gibbs free energy barrier, and ΔrG represents the Gibbs free energy change of the reaction.

The addition reaction of the ·OH with the P=S bond in CPY first forms PRC1, releasing 6.62 kcal mol^−1^ of heat. The configuration of PRC1 is shown in Figure S1. Then, it proceeded through transition state TS1-1(OH), which had a very low Gibbs free energy barrier of only 1.96 kcal mol^−1^. The ΔrG is −14.34 kcal·mol^−1^, indicating that the reaction could occur spontaneously.

Since the pyridine ring has six delocalized π electrons, the ·OH could attach to atoms C_2_, C_3_, C_4_, C_5_, C_6_, and N_7_. By comparing the Gibbs free energy barriers, it is found that the addition to the C atoms had relatively low barriers ranging from 2.23 to 3.46 kcal·mol^−1^, and the free energy changes were negative, leading to spontaneous reactions. However, the addition to the N_7_ atom had a higher ΔG^≠^ of 18.87 kcal·mol^−1^ and a positive ΔrG of 15.30 kcal·mol^−1^, indicating that it could not occur spontaneously at room temperature.

In addition, the ·OH could also abstract H atoms from CPY, including the H atoms on the two ethyl groups and the H atom on the pyridine ring. All the abstraction reactions have been calculated (Figure 3). By comparing the ΔG^≠^ and ΔrG, it be observed that the barriers for the H atoms on the ethyl groups are relatively low, ranging from 1.42 to 4.84 kcal·mol^−1^, while the barrier for the pyridine ring was relatively high at 13.72 kcal·mol^−1^. All the energy changes were negative, indicating that spontaneous reactions could be occurred at room temperature.

Overall, from a thermodynamic perspective, except for the reaction with N_7_ atoms having a high ΔG^≠^ and being non-spontaneous, the other addition and abstraction reactions could occur spontaneously.

### 3.3. The Reaction between CPY and the SO_4_^•−^

**Figure 4:**
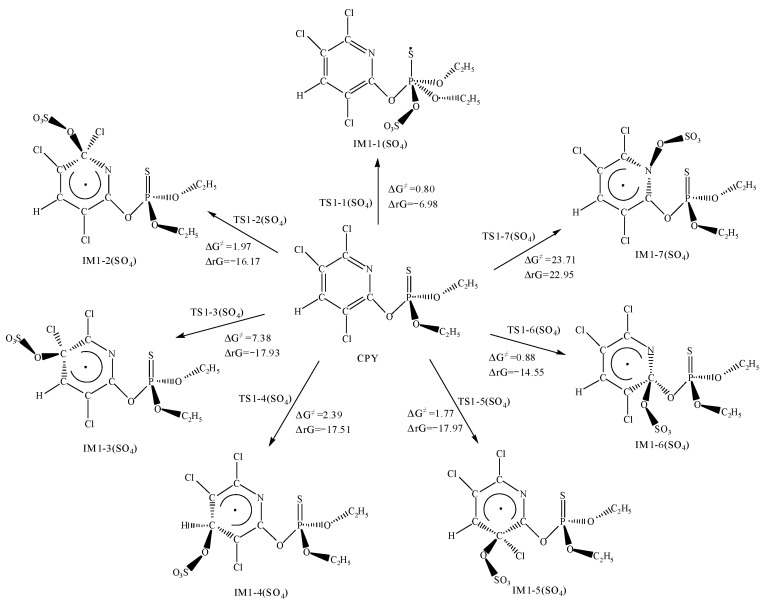
The Gibbs free energy barrier ΔG^≠^ (kcal·mol^−1^) and the Gibbs free energy change ΔrG (kcal·mol^−1^) at 298 K for SO_4_^•−^ addition pathways with CPY.

**Figure 5:**
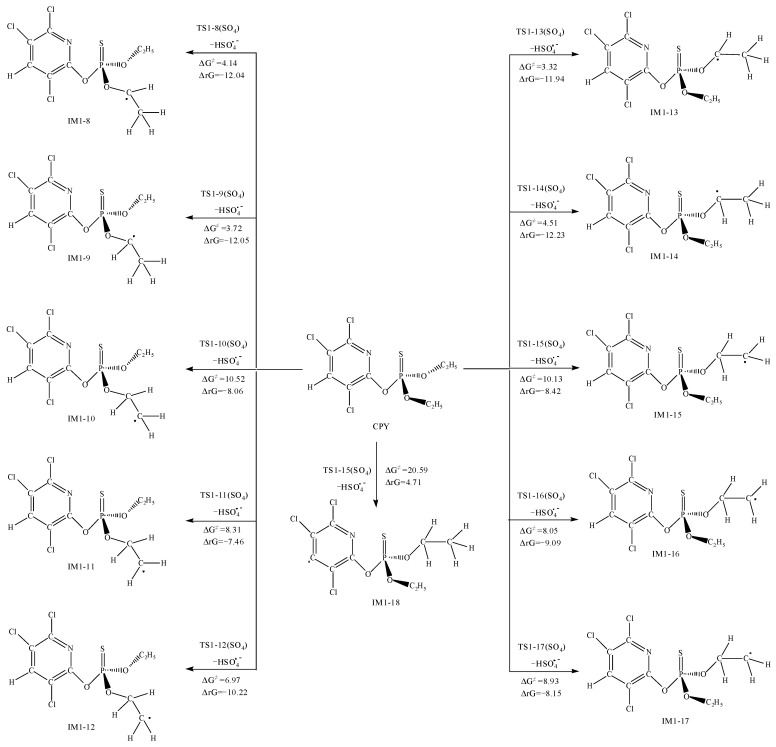
The Gibbs free energy barrier ΔG^≠^ (kcal·mol^−1^) and the Gibbs free energy change ΔrG (kcal·mol^−1^) at 298 K for SO_4_^•−^ abstraction pathways with CPY.

The mechanism of the reaction initiated by SO_4_^•−^ was similar to that of CPY with ·OH, including addition reactions and H abstraction reactions. The ΔG^≠^ and ΔrG for SO_4_^•−^ addition pathways and abstraction pathways with CPY are shown in Figure 4 and Figure 5, respectively. The optimized chemical conformations of TS for CPY with SO_4_^•−^ are shown in Figure S2.

For the addition reaction of SO_4_^•−^, the most likely site is the P=S bond, with a ΔG^≠^ of 0.80 kcal·mol^−1^, and releasing 6.98 kcal·mol^−1^ of energy. Additionally, the addition reactions to the C atoms in the pyridine ring had low barriers ranging from 0.88 to 7.38 kcal·mol^−1^. However, the addition reaction to the N_7_ atom had a high ΔG^≠^ of 23.71 kcal·mol^−1^ and a positive ΔrG, indicating that it could not occur spontaneously at room temperature.

Furthermore, as shown in Figure 5, SO_4_^•−^ can abstract H atoms connected to the C atoms, forming HSO_4_^•−^. By comparing the ΔG^≠^ for the H abstraction reactions at different positions, the H atom connected to the C atom bonded with O was most easily abstracted, with barriers ranging from 3.32 to 4.51 kcal·mol^−1^. Next is the abstraction from the methyl group, with a barrier between 6.97 to 10.52 kcal·mol^−1^. Moreover, the H atom on the pyridine ring was the most difficult to abstract, with a ΔG^≠^ of 20.59 kcal·mol^−1^ and a positive ΔrG, indicating it could not occur spontaneously at room temperature.

Overall, from the view of thermodynamics, except for the addition reaction to N_7_, the abstraction of H atoms on the pyridine ring had high ΔG^≠^, which was not easy to occur spontaneously at room temperature, the other addition and abstraction reactions could occur spontaneously. For a further exploration and evaluation for the likelihood of the reactions and the contribution of each reaction pathway, kinetic calculations for each elementary reaction comparing the magnitudes of the rate constants to determine the optimal pathway were performed.

### 3.4. Rate Constants Calculation

The rate constants (*k*) for the initiation reactions of CPY by ·OH and SO_4_^•−^ were calculated at 298 K, including all possible addition and abstraction reactions. The calculated results are shown in Table 1, where *k*_add_ represents the rate constants for addition reactions of ·OH or SO_4_^•−^, k_abs_ represents the rate constants for abstraction reactions of ·OH or SO_4_^•−^, and *Γ* represents the branching ratio. The formula for calculating *Γ* is given by *Γ* = *k_i_*/*k*_total_, where *k*_total_ is the sum of the rate constants for addition and abstraction reactions of ·OH and SO_4_^•−^ with CPY.

When the temperature is 298 K, the *Γ* of different reaction pathways are as follows: the abstraction reaction between SO_4_^•−^ and C_9_ atom had the highest *Γ* (45.43%), followed by the addition reaction between P=S bond and ·OH (20.12%); the abstraction reaction between SO_4_^•−^ and C_8_ atom (11.36%), and the abstraction reactions between ·OH and C_8_ atom, and ·OH and C_8_ atom (9.78% and 5.05%, respectively). Lastly, there were the abstraction reactions between the ·OH and C_13_ atom, and between the SO_4_^•−^ and C_13_ atom, accounting for 4.39% and 1.75%, respectively. The rest of the reaction pathways could be considered negligible. The calculated total rate constants for the reactions of CPY with ·OH and SO_4_^•−^ were 6.32 × 10^8^ and 9.14 × 10^8^ M^−1^· s^−1^ at 298 K, which was consistent with the experimental values of 4.42 × 10^9^ and 4.5 × 10^9^ M^−1^·s^−1^ [47]. Results indicated that the calculated results and subsequent theoretical analysis were reliable and valuable.

Pathways with high branching ratios are favorable starting channels [31]. Thus, the corresponding intermediates IM1-1(OH), IM1-8, IM1-9, and IM1-13 were selected as the subjects of study to explore the subsequent reactions of CPY.

### 3.5. Subsequent Reactions

#### 3.5.1. Subsequent Reactions of IM1-1(OH)

**Figure 6:**
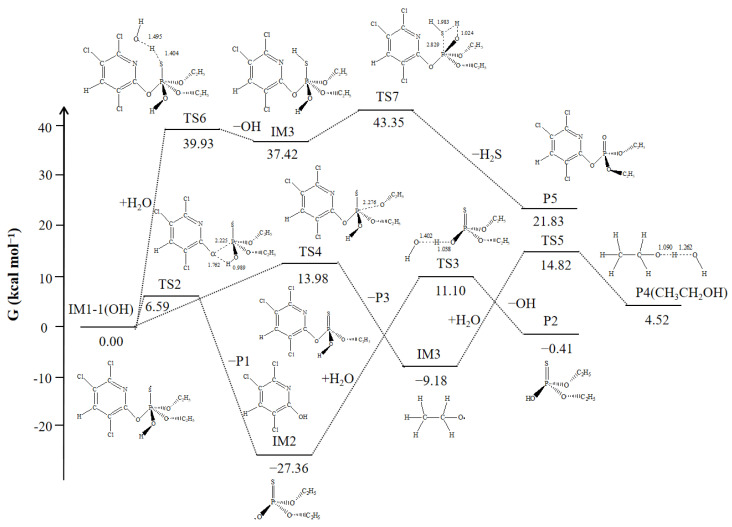
The reaction path profiles of the subsequent reactions of IM-1(OH).

Due to the attack of ·OH, the intermediate IM1-1(OH) became highly reactive with unpaired electrons, thus becoming reactive and capable of generating stable products through its own bond-breaking reactions and H atom abstraction from water (H_2_O). The subsequent reactions were illustrated in Figure 6, which involved three pathways. The first pathway involved the cleavage of the P_1_–O_21_ bond, resulting in the formation of products P1 (3,5,6-trichloro-2-pyridynol) and the IM2 radical. The IM2 radical further abstracted an H atom from H_2_O to produce the stable product P2 (O,O-diethyl phosphorothioate). The second pathway involved the cleavage of either the P_1_–O_23_ or P_1_–O_26_ bond. Since these two bond cleavage modes were symmetrical, only the cleavage of the P_1_–O_23_ bond was described here. The process led to the formation of P3 and the IM3 radical, which stabilized into product P4 (ethanol) through H atom abstraction reactions. The third pathway involved a bimolecular reaction, where the S atom with unpaired electrons abstracted an H atom from H_2_O, followed by the elimination of H_2_S to produce P5 (chlorpyrifos oxon). All products except P4 have been detected by HPLC-TOF-MS/MS in the combination system of microwave and persulfate [27]. By comparing the energy barriers of these three pathways, the cleavage of the P_1_=O_21_ bond was the most favorable, making it the optimal pathway, which was consistent with the experimental detection of a significant amount of P1 [26,27].

#### 3.5.2. Subsequent Reactions of IM1-8, IM1-9, and IM1-13

**Figure 7:**
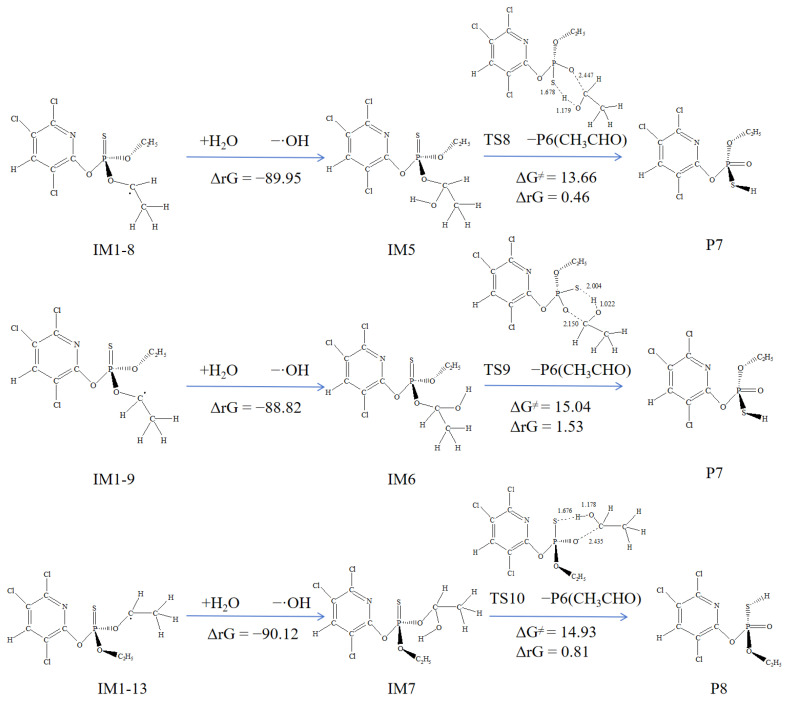
Subsequent reactions of IM1-8, IM1-9, and IM1-13 with the Gibbs free energy barrier ΔG^≠^ (kcal·mol^−1^) and the Gibbs free energy change ΔrG (kcal·mol^−1^).

The reactions of M1-8, IM1-9, and IM1-13 were very similar, as they all involve barrierless addition with large amount of ·OH present in the solution. As shown in Figure 7, the ΔrG of this process was highly negative, making it easily spontaneous. Subsequently, P7, P8, and P6 (acetaldehyde) are obtained through dealkylation reactions. The ΔG^≠^ for these processes range from 16.61 to 18.90 kcal·mol^−1^, indicating that these reactions are possibly occurred, especially under high-temperature conditions. Corresponding products were also detected in related experimental studies [27].

In conclusion, in the persulfate-based AOP system, both ·OH and SO_4_^•−^ played important roles in the degradation of CPY. The main initiating reactions involved the addition of ·OH to the P=S bond and the H atoms abstraction reactions with the C atoms connected to the P atom. The addition products undergo P_1_–O_21_ bond cleavage between the pyridine ring and the P atom, leading to the formation of P1. The abstraction products mainly underwent dealkylation reactions to complete the degradation processes.

### 3.6. Toxicity Assessment

In this study, the computational toxicity software ECOSAR V2.2 and TEST V5.1.1 were used to assess the eco-toxicity of CPY and its degradation products. Although conducting toxicity experiments is irreplaceable, computational toxicology can conveniently provide a large number of toxicity characteristic values at the screening level. It is widely used in ecological toxicity assessment due to its convenience, speed, cost-effectiveness, and independence from specific experimental animals. Liu et al. used the ECOSAR model to predict the acute and chronic toxicity changes of CPY and its degradation products with ferrate [48]. The results showed that most of the products generated in the later stage of the reaction were classified as non-toxic to all tested organisms. And the dechlorination, ·OH substitution, C–O bond cleavage, and P=S bond oxidation are highly effective in detoxifying CPY. Shah et al. used the ECOSAR toxicity model on the photocatalytic of CPY to predict the toxicity of its transformation products [25]. The formation of toxic intermediate compounds helps remind researchers to evaluate the toxicity of CPY and the transformation products, and the formation of non-toxic acetate esters as the final product indicates that the treatment technique has significant capabilities for detoxifying CPY.

The ECOSAR ecological toxicity model was used to predict the acute toxicity of CPY and its degradation products to fish, dephnia, and green algae. Table S2 lists the classification criteria for acute and chronic toxicity, while Table S3 lists the acute and chronic toxicity values of CPY and its degradation products. As can be seen, the calculated LC_50_ value of CPY to fish and daphnia are 38 and 0.19 μg·L^−1^, respectively, which are in good agreement with the experimental measured results of 25.78 μg·L^−1^ for mozambique tilapia and 0.235 to 0.512 μg·L^−1^ for daphnia [49,50]. The calculated EC_50_ value of CPY to green algae is 176 μg·L^−1^, which is slightly lower than the 769 μg·L^−1^ value measured in the experiment [51]. This also demonstrates the accuracy of the computational toxicity prediction method.

**Figure 8:**
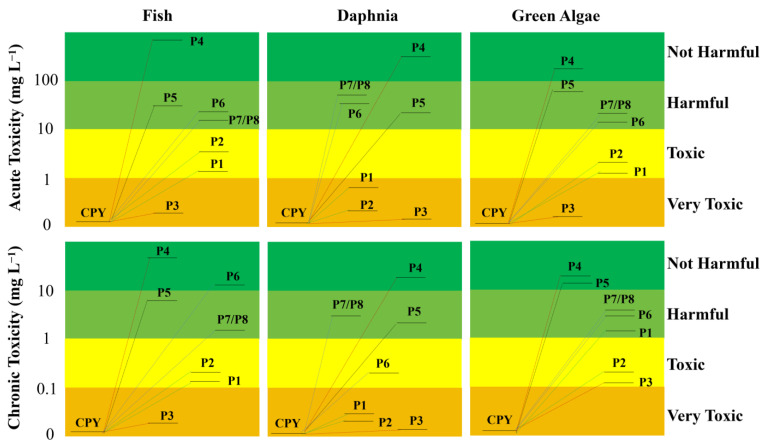
Acute and chronic toxicity of CPY and its transformation products to aquatic organisms.

Based on the toxicity classification of the Globally Harmonized System (GHS), CPY is defined to be highly toxic compounds [52]. As shown in Figure 8, the toxicity evolution diagram was plotted based on the toxicity classification of the GHS. The acute and chronic toxicity changes indicated that the acute and chronic toxicity of the main degradation products to fish, dephnia, and green algae were lower than that of the parent CPY. However, P1, P2, and P3 still exhibited toxic or highly toxic levels. Therefore, a careful assessment of the potential environmental risks posed by the degradation products is still needed.

The TEST software was used to predict the developmental toxicity and mutagenicity of CPY and its products. Table S4 shows that CPY and its products were non-mutagenic. Developmental toxicity could disrupt the homeostasis, normal growth, differentiation, and development of organisms, suggesting that P3, P5, P7, and P8 may have adverse effects on biological development. However, eco-toxicity and health effects were not only dependent on exposure levels but also on bioaccumulation. The bioaccumulation factors of CPY and its products were much lower than 5000, indicating low bioaccumulation [53]. Low bioaccumulation would not further affect the eco-toxicity and health effects of the compounds. This also suggests that the persulfate-based AOP system could be an effective method to eliminate chlorpyrifos contamination in water environment.

## 4. Conclusions

In this study, the mechanism of CPY degradation in water environment using persulfate–based AOPs was investigated using the DFT method. The ecological toxicity of CPY and its products was also evaluated. Results indicated that the ·OH and SO_4_^•−^ generated from persulfate activation could undergo addition and abstraction reactions with CPY. Thermodynamic and kinetic research indicated that the addition of ·OH to the P=S bond and the abstraction of H atom from the C connected to P atom by both types of active radicals were the main initiating reactions. The reaction rate constants of CPY with ·OH and SO_4_^•−^ were 6.32 × 10^8^ and 9.14 × 10^8^ M^−1^·s^−1^ at room temperature, respectively, which were strongly consistent with experimental data. The addition products underwent the cleavage reaction of the P–O bond between the pyridine ring and the P atom, leading to the formation of the main product of P1. The abstraction products mainly underwent dealkylation reactions to complete the degradation. The acute and chronic toxicities of the main eight degradation products to fish, dephnia, and green algae were found to be lower than those of the parent CPY. However, products of P1, P2, and P3 still exhibited toxic or highly toxic levels, which may pose risks to human health. However, due to their low bioaccumulation, their ecological toxicity and health effects may be limited. Overall, these findings provide theoretical support for the application of persulfate-based AOP system for the removal of eliminating chlorpyrifos contamination in water environment.

## Figures and Tables

**Table 1:** The total and separated rate constant (*k*) and branch ratio (*Γ*) for in the reaction of CPY with ·OH and SO_4_^•−^ at 298 K in water system.

Reaction	k_298K_ (M^−1^ ·s^−1^ )	*Γ*(%)	Reaction	k_298K_ (M^−1^ ·s^−1^)	*Γ*(%)
CPY+·OH→vdW→IM1-1	3.11 × 10^8^	20.12	CPY+SO_4_^•−^→IM1-1(SO_4_)	3.89 × 10^1^	0
CPY+·OH→IM1-2(OH)	2.03 × 10^3^	0	CPY+SO_4_^•−^→IM1-2(SO_4_)	9.62 × 10^2^	0
CPY+·OH→IM1-3(OH)	5.93 × 10^2^	0	CPY+SO_4_^•−^→IM1-3(SO_4_)	1.61 × 10^−1^	0
CPY+·OH→IM1-4(OH)	5.71 × 10^3^	0	CPY+SO_4_^•−^→IM1-4(SO_4_)	3.30	0
CPY+·OH→IM1-5(OH)	8.12 × 10^3^	0	CPY+SO_4_^•−^→IM1-5(SO_4_)	8.02 × 10^2^	0
CPY+·OH→IM1-6(OH)	2.60 × 10^3^	0	CPY+SO_4_^•−^→IM1-6(SO_4_)	7.58	0
CPY+·OH→IM1-7(OH)	2.30 × 10^−8^	0	CPY+SO_4_^•−^→IM1-7(SO_4_)	1.03 × 10^−7^	0
$k_{add}^{OH}$	3.11 × 10^8^	20.12	$k_{add}^{{SO}_{4}}$	1.81 × 10^3^	0
CPY+·OH→IM1-8+H_2_O	1.51 × 10^8^	9.78	CPY+SO_4_^•−^→IM1-8+HSO_4_^·-^	1.76 × 10^8^	11.36
CPY+·OH→IM1-9+H_2_O	7.80 × 10^7^	5.05	CPY+SO_4_^•−^→IM1-9+HSO_4_^·-^	7.02 × 10^8^	45.43
CPY+·OH→IM1-10+H_2_O	1.08 × 10^7^	0.70	CPY+SO_4_^•−^→IM1-10+HSO_4_^·-^	2.94	0
CPY+·OH→IM1-11+H_2_O	3.52 × 10^5^	0.02	CPY+SO_4_^•−^→IM1-11+HSO_4_^·-^	8.77 × 10^5^	0.06
CPY+·OH→IM1-12+H_2_O	4.47 × 10^6^	0.29	CPY+SO_4_^•−^→IM1-12+HSO_4_^·-^	3.35 × 10^6^	0.22
CPY+·OH→IM1-13+H_2_O	6.78 × 10^7^	4.39	CPY+SO_4_^•−^→IM1-13+HSO_4_^·-^	2.71 × 10^7^	1.75
CPY+·OH→IM1-14+H_2_O	3.33 × 10^6^	0.21	CPY+SO_4_^•−^→IM1-14+HSO_4_^·-^	4.52 × 10^6^	0.29
CPY+·OH→IM1-15+H_2_O	3.04 × 10^6^	0.20	CPY+SO_4_^•−^→IM1-15+HSO_4_^·-^	8.36 × 10^3^	0
CPY+·OH→IM1-16+H_2_O	2.96 × 10^5^	0.02	CPY+SO_4_^•−^→IM1-16+HSO_4_^·-^	2.03 × 10^3^	0
CPY+·OH→IM1-17+H_2_O	1.68 × 10^6^	0.11	CPY+SO_4_^•−^→IM1-17+HSO_4_^·-^	8.14 × 10^3^	0
CPY+·OH→IM1-18+H_2_O	2.67 × 10^−3^	0	CPY+SO_4_^•−^→IM1-18+HSO_4_^·-^	1.75 × 10^−4^	0
$k_{abs}^{OH}$	3.21 × 10^8^	20.77	$k_{abs}^{{SO}_{4}}$	9.14 × 10^8^	59.11

## Data Availability

Data are contained within the article.

## Supplementary Materials

The following supporting information can be downloaded at: https://www.mdpi.com/article/10.3390/toxics12030207/s1, Figure S1: The optimized chemical conformations of TS for CPY with ·OH; Figure S2: The optimized chemical conformations of TS for CPY with SO_4_^•−^; Table S1: The bond lengths of the calculated values and the experimental values of CPY; Table S2: The grading standards of the acute and chronic toxicity. The unit is mg·L^−1^.; Table S3: The toxicity value of the main transformation intermediates and products in the degradation of CPY. The unit is mg·L^−1^.; Table S4: Estimated health effects of CPY and its transformation intermediates and products during the degradation process.
