# Supplementary material for: Persulfate–Based Advanced Oxidation Process for Chlorpyrifos Degradation: Mechanism, Kinetics, and Toxicity Assessment
Source: Toxics. 2024 Mar 9;12(3):207. doi: 10.3390/toxics12030207 (PMC10974677; doi:10.3390/toxics12030207)
Supplement: Supplementary file 1 [file toxics-12-00207-s001.zip › toxics-2902824-supplementary.pdf]

Persulfate–Based Advanced Oxidation Process for Chlorpyrifos Degradation: Mechanism, Kinetics, and Toxicity Assessment

Youxin Xu [1,2], Chenxi Zhang [2], Haobing Zou [2], Guangrong Chen [2], Xiaomin Sun [3], Shuguang Wang [1,4,*] and Huifang Tian [1,*]

1 Institute of Environmental Biotechnology and Functional Materials, School of Environmental Science and Engineering, Shandong University, Qingdao 266237, China; xuyouxin@wfust.edu.cn
2 Shandong Engineering Laboratory for Clean Utilization of Chemical Resources, Weifang University of Science and Technology, Weifang 262700, China; sdzhangcx@gmail.com (C.Z.); A15910058268@outlook.com (H.Z.); guangrongchen2004@outlook.com (G.C.)
3 Environment Research Institute, Shandong University, Qingdao 266237, China; sxmwch@sdu.edu.cn
4 Sino-French Research Institute for Ecology and Environment (ISFREE), Shandong University,
Qingdao 266237, China
* Correspondence: wsg@sdu.edu.cn (S.W.); hftian@sdu.edu.cn (H.T.)

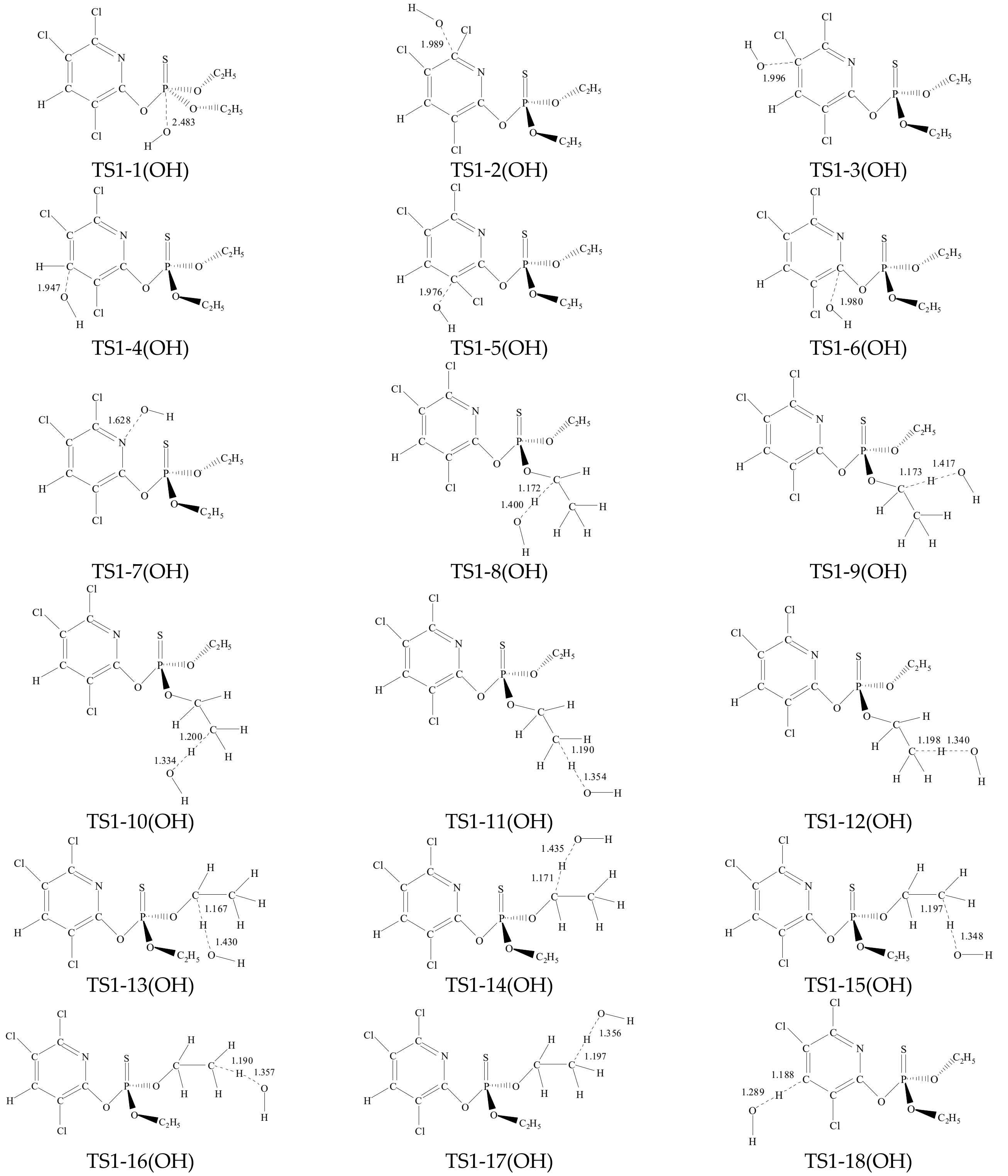

Figure S1. The optimized chemical conformations of TS for CPY with OH.

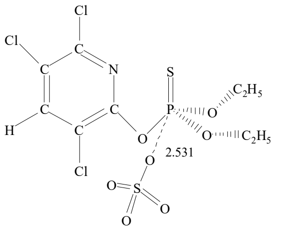
TS1-1(SO4)

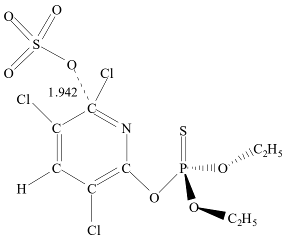
TS1-2(SO4)

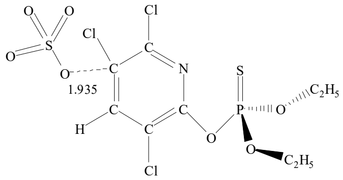
TS1-3(SO4)

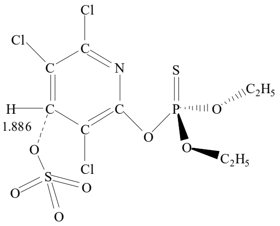
TS1-4(SO4)

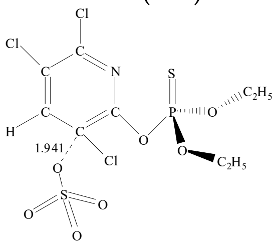
TS1-5(SO4)

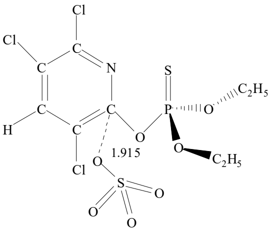
TS1-6(SO4)

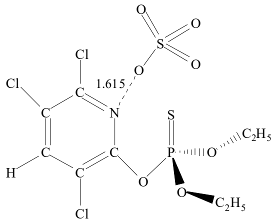
TS1-7(SO4)

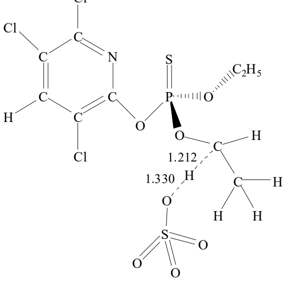
TS1-8(SO4)

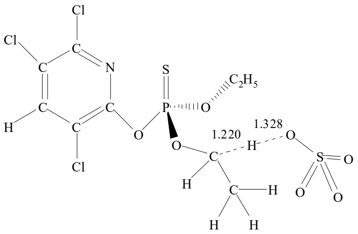
TS1-9(SO4)

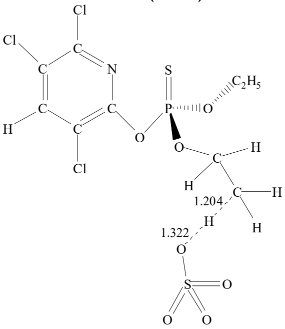
TS1-10(SO4)

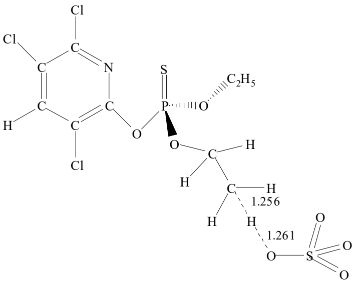
TS1-11(SO4)

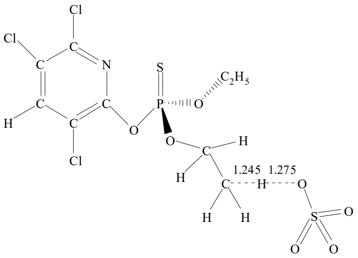
TS1-12(SO4)

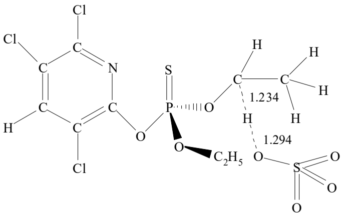
TS1-13(SO4)

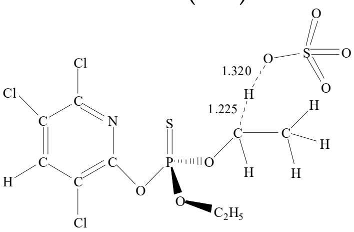
TS1-14(SO4)

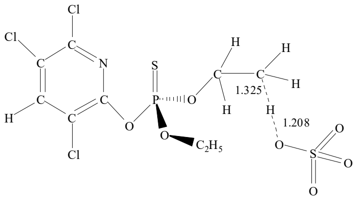
TS1-15(SO4)

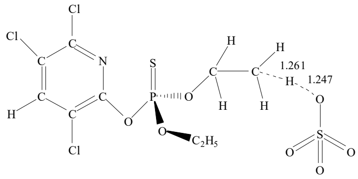
TS1-16(SO4)

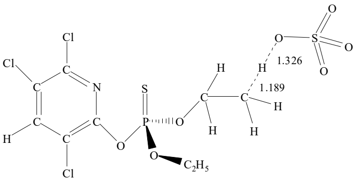
TS1-17(SO4)

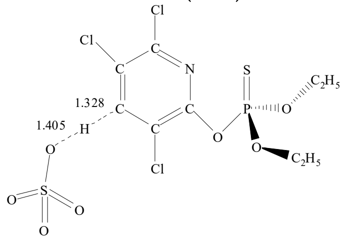
TS1-18(SO4)

Figure S2. The optimized chemical conformations of TS for CPY with $SO_4^{\bullet-}$.

Table S1. The bond lengths of the calculated values and the experimental values of CPY.

Bond	Calculated values (Å)	Experimental values (Å)	Relative errors
P1-O21	1.611	1.647	2.23%
P1-O23	1.562	1.593	1.98%
P1-O26	1.538	1.59	3.38%
P1=S22	1.898	1.921	1.21%
O23-O24	1.426	1.447	1.47%
C24-C25	1.44	1.512	5.00%
O26-C27	1.39	1.442	3.74%
C2-C3	1.37	1.394	1.75%
C3-C4	1.39	1.394	0.29%
C4-C5	1.37	1.384	1.02%
C5-C6	1.38	1.403	1.67%
C6-N7	1.308	1.319	0.84%
N7-C2	1.32	1.321	0.08%
C6-O21	1.364	1.347	1.25%

Table S2. The grading standards of the acute and chronic toxicity. The unit is mg·L^{-1}.

Classification	Acute toxicity(1)	Chronic toxicity(2)
Very toxic	LC_{50}≤1 or EC_{50}≤1	ChV≤0.1
Toxic	1<LC_{50}≤10 or 1<EC_{50}≤10	0.1<ChV≤1
Harmful	10<LC_{50}≤100 or 10<EC_{50}≤100	1<ChV≤10
Not harmful	LC_{50}>100 or EC_{50}>100	ChV>10

Criteria set by the Chinese hazard evaluation guidelines for new chemical substances (HJ/T 154–2004). Criteria set by the European Union (described in Annex VI of Directive 67/548/EEC).

Table S3. The toxicity value of the main transformation intermediates and products in the degradation of CPY. The unit is mg·L^{-1}.

Molecular Structure		ChV			LC_{50}		
		Fish	Daphnid	Algae	Fish	Daphnid	Algae
CPY	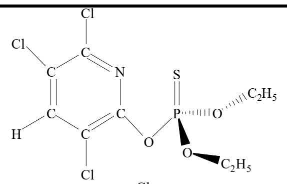	0.0065	0.000037	0.084	0.038	0.00019	0.176
P1	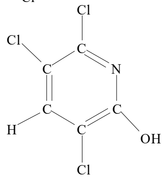	0.132	0.021	1.35	1.10	0.836	1.816
P2	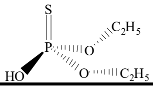	0.359	0.00013	0.366	5.31	0.010	3.88

P3	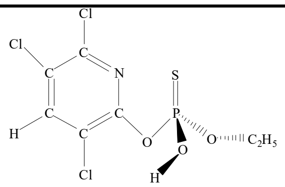	0.015	0.000051	0.120	0.102	0.00044	0.338
P4	CH_3CH_2OH	245	83.1	81.9	3170	1480	486
P5	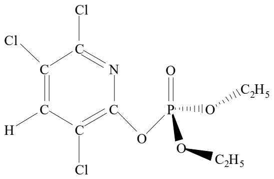	9.05	4.67	24.2	30.0	28.2	85.4
P6	CH_3CHO	12.1	0.242	4.20	29.0	32.6	15.1
P7/P8	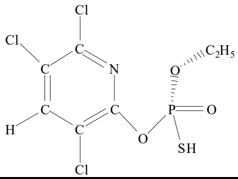	1.34	6.66	6.42	19.5	44.1	20.0

Table S4. Estimated health effects of CPY and its transformation intermediates and products during the degradation process.

	Molecular Structure	Bioaccumulation factor	Developmental Toxicity	Mutagenicity
CPY	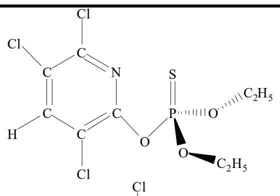	380.62	Developmental toxicant	Mutagenicity Negative
P1	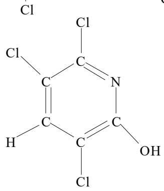	73.23	Developmental NON-toxicant	Mutagenicity Negative
P2	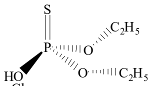	2.66	Developmental NON-toxicant	Mutagenicity Negative
P3	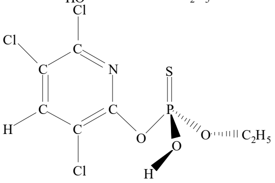	143.97	Developmental toxicant	Mutagenicity Negative
P4	CH_3CH_2OH	1.26	Developmental NON-toxicant	Mutagenicity Negative
P5	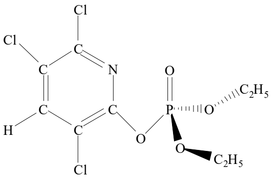	109.09	Developmental toxicant	Mutagenicity Negative
P6	CH_3CHO	N/A	Developmental NON-toxicant	Mutagenicity Negative
P7/P8	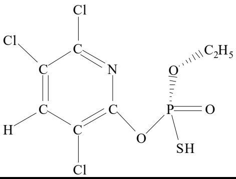	N/A	Developmental toxicant	Mutagenicity Negative
